# When Everything Becomes Bigger: Big Data for Big Poultry Production

**DOI:** 10.3390/ani13111804

**Published:** 2023-05-30

**Authors:** Giovanni Franzo, Matteo Legnardi, Giulia Faustini, Claudia Maria Tucciarone, Mattia Cecchinato

**Affiliations:** Department of Animal Medicine, Production and Health (MAPS), University of Padua, 35020 Legnaro, Italy; matteo.legnardi@unipd.it (M.L.); giulia.faustini.1@phd.unipd.it (G.F.); claudiamaria.tucciarone@unipd.it (C.M.T.); mattia.cecchinato@unipd.it (M.C.)

**Keywords:** poultry, big data, sensors, machine learning, deep learning, sequencing, IoT, production, pathogens

## Abstract

**Simple Summary:**

In future decades, the demand for poultry meat and eggs is predicted to considerably increase in pace with human population growth. Although this expansion clearly represents a remarkable opportunity for the sector, it conceals a multitude of challenges related to pollution and land erosion, competition for limited resources between animal and human nutrition, animal welfare concerns, limitations on the use of growth promoters and antimicrobial agents, and increasing risks of animal infectious diseases and zoonoses. The increase in poultry production must be achieved mainly through optimization and increased efficiency. The increasing ability to generate large amounts of data (“big data”)—coupled with the availability of tools and computational power to store, share, integrate, and analyze data with automatic and flexible algorithms—offers an unprecedented opportunity to develop tools to maximize farm profitability, reduce socio-environmental impacts, and increase animal and human health and welfare. The present work reviews the application of sensor technologies, specifically, the principles and benefits of advanced statistical techniques and their use in developing effective and reliable classification and prediction models to benefit the farming system. Finally, recent progress in pathogen genome sequencing and analysis is discussed, highlighting practical applications in epidemiological tracking and control strategies.

**Abstract:**

In future decades, the demand for poultry meat and eggs is predicted to considerably increase in pace with human population growth. Although this expansion clearly represents a remarkable opportunity for the sector, it conceals a multitude of challenges. Pollution and land erosion, competition for limited resources between animal and human nutrition, animal welfare concerns, limitations on the use of growth promoters and antimicrobial agents, and increasing risks and effects of animal infectious diseases and zoonoses are several topics that have received attention from authorities and the public. The increase in poultry production must be achieved mainly through optimization and increased efficiency. The increasing ability to generate large amounts of data (“big data”) is pervasive in both modern society and the farming industry. Information accessibility—coupled with the availability of tools and computational power to store, share, integrate, and analyze data with automatic and flexible algorithms—offers an unprecedented opportunity to develop tools to maximize farm profitability, reduce socio-environmental impacts, and increase animal and human health and welfare. A detailed description of all topics and applications of big data analysis in poultry farming would be infeasible. Therefore, the present work briefly reviews the application of sensor technologies, such as optical, acoustic, and wearable sensors, as well as infrared thermal imaging and optical flow, to poultry farming. The principles and benefits of advanced statistical techniques, such as machine learning and deep learning, and their use in developing effective and reliable classification and prediction models to benefit the farming system, are also discussed. Finally, recent progress in pathogen genome sequencing and analysis is discussed, highlighting practical applications in epidemiological tracking, and reconstruction of microorganisms’ population dynamics, evolution, and spread. The benefits of the objective evaluation of the effectiveness of applied control strategies are also considered. Although human-artificial intelligence collaborations in the livestock sector can be frightening because they require farmers and employees in the sector to adapt to new roles, challenges, and competencies—and because several unknowns, limitations, and open-ended questions are inevitable—their overall benefits appear to be far greater than their drawbacks. As more farms and companies connect to technology, artificial intelligence (AI) and sensing technologies will begin to play a greater role in identifying patterns and solutions to pressing problems in modern animal farming, thus providing remarkable production-based and commercial advantages. Moreover, the combination of diverse sources and types of data will also become fundamental for the development of predictive models able to anticipate, rather than merely detect, disease occurrence. The increasing availability of sensors, infrastructures, and tools for big data collection, storage, sharing, and analysis—together with the use of open standards and integration with pathogen molecular epidemiology—have the potential to address the major challenge of producing higher-quality, more healthful food on a larger scale in a more sustainable manner, thereby protecting ecosystems, preserving natural resources, and improving animal and human welfare and health.

## 1. Introduction

Poultry production plays a critical role in the global economy. Pressure on the agricultural system will increase with the continuing expansion of the human population. By the end of 2050, the demand for poultry meat is estimated to double, and the demand for eggs is estimated to increase by 40%, representing an important source of highly valuable and inexpensive protein [1,2]. Beyond industrial farming, particularly in the small-scale village context, chicken farming can significantly contribute to poverty alleviation through income generation and household food security [3,4]. Although the increase in poultry demand represents a great opportunity for the industry, it also conceals a multitude of challenges. Pollution and land erosion, competition for limited resources between animal and human nutrition, animal welfare concerns, limitations in the use of antimicrobial agents, and increasing risks and impacts of animal infectious diseases and zoonoses are only some of the topics that have concerned authorities and the public [5]. Whether real or perceived, these aspects pose severe limitations to further expansion of traditional poultry production. A clear solution would be the improvement of production efficiency. Accurate, prompt, and dynamic collection, integration, and analysis of large amounts of data have been key to the success of many productive activities and have become an essential part of our lives. Technologies such as sensors, cloud computing, machine learning (ML), and artificial intelligence (AI) are transforming several industries. Although data collection is already routinely applied in certain agriculture and farming realities, including poultry farming, skepticism persists regarding this approach [6,7]. Because the production of poultry—one of the fastest-growing production species—uses highly similar management strategies worldwide and high levels of integration, it offers ideal conditions for the application of new technological developments. Moreover, most animal farmers now have access to modern technologies—such as high-speed internet, smartphones, and inexpensive computing power—which were unavailable a decade ago [8,9]. Unfortunately, many strategies for big data integration, sharing, and analysis remain at early stages of development. Hardware sensors (such as cameras or vision sensors; infrared thermal imaging sensors; temperature sensors; radio frequency identification tags; accelerometers; motion sensors; or microphones) can generate an astonishing amount of information (big data) [1,10]. Similarly, instances of progress in sequencing technologies allow for a continuous increase in host, microorganism and pathogen genomes and gene expression profiles. Advanced AI and ML algorithms can be integrated in the data analysis process and make use of these extensive data to analyze, predict, and notify farmers of abnormal occurrences, identifying patterns and suggesting solutions to pressing problems in modern animal farming, driving the strategies to improve the sector’s profitability [8,11]. The definition of big data is somewhat elusive and is often described in terms of three “Vs”: volume, velocity, and variety [12]. Volume refers to a large amount of data, velocity refers to high speed of data generation, and variety refers to data coming from different sources and/or consisting of different types. However, with the continuing growth of the field, the definition has also become “bigger”, and 42 Vs of big data and data science have been proposed [13]. Therefore, a complete discussion of the field of big data and its applications in poultry farming is impossible in this brief text. Consequently, a limited number of illustrative topics have been selected to be herein discussed, pertaining to data generation, organization, and analysis. In particular, the main focus is on the collection and application of productive/behavioral data detectable on the farm and genetic data from microorganisms and pathogens.

## 2. Big Data on the Farm

### 2.1. Sensors and Data Generation

In response to the above-mentioned challenges, changes in farming strategies and the implementation of new smart management technologies are highly relevant. These include precision livestock farming practices, in addition to other technologies associated with the collection and use of farm-generated data. Precision livestock farming is the management of livestock production benefitting from automatic data acquisition, access, and processing [14,15]. In intensive poultry production, many factors, such as stocking density, environmental deterioration, unsuitable social environments, thermal stress, or difficulties in accessing essential resources, can be major sources of stress leading to welfare deterioration and reduced performance [16,17]. The collection of environmental variables, such as temperature, air velocity, ventilation rate, litter quality, humidity, and gas concentration, has clear benefits in poultry welfare, mortality, and performance, thereby helping producers reach the desired level of production [1] (Table 1).

However, although environmental and animal data can be acquired by a multitude of sensors [55], such data, except those for temperature, are not commonly collected in most commercial poultry farms. Temperature, relative humidity, carbon dioxide, and ammonia level monitoring have been effectively used to predict broiler weight as many as 72 h in advance [53]. Such systems can enable early interventions and the achievement of target weight. Integration with other non-invasive surveillance technologies developed and implemented in poultry houses, including those for health, welfare, and feeding, would enable more data to be incorporated into predictive production models, thus potentially enhancing their capabilities. Acoustic sensors have been developed for exploitation of birds’ acoustic communications for their social interactions and alarm signaling; some can also be considered reliable stress indicators [15]. Using acoustic parameters such as vocalization frequency has enabled detection of episodes of food deprivation or the inadequacy of the thermal environment in broilers and laying hens [60,61]. Similarly, higher rates of squawks and total vocalizations have been observed in laying hen flocks with feather pecking problems [62]. Detection of infections with pathogenic microorganisms is also possible with this technology. The frequency of rales produced by chickens infected with infectious bronchitis virus (IBV) has been shown experimentally to enable detection of infections before clinical signs are evident [24,25]. Sadeghi et al. have recorded broiler vocalizations in healthy and Clostridium-perfringens-infected birds. An artificial neural network model was able to differentiate between infected and healthy birds with an accuracy of 66.6% on day 2 after infection and 100% on day 8 [26]. Air sensors in the poultry industry can now predict the onset of coccidiosis by monitoring volatile organic compounds in the air that increase as the number of infected birds increases, thus enabling much earlier detection of infection spread than would be achievable by farmers or veterinarians [28]. Alerted farmers would be able to take timely measures to prevent further spread of the infection. Such systems could save several animal lives and prevent financial losses [10]. Similarly, wearable sensors such as accelerometers have been demonstrated as being useful in identifying influenza viral infection in chickens, by detecting changes in physiology and movement patterns [18]. Although this sensing equipment can prevent economic losses and welfare issues due to disease spread, it would be unpractical and too expensive to fit all individuals in a typically-large poultry flock with surveillance equipment. However, sensors could be used in a subpopulation of sentinel birds, and may be effective for prevention or early detection, at least in high-risk areas [15]. Therefore, smart poultry management practices can mitigate the risks of infection and disease, and the consequent health threat to both animals and humans, through prompt diagnosis and detection at the point of care (i.e., performing a medical diagnostic testing in an area where a patient can receive care) [63]. Rapid detection systems continuously monitoring poultry for disease can complement pre-existing approaches to infectious disease detection and diagnosis. The combination of early warning systems and rapid diagnosis could enable immediate action to be taken, preventing subsequent spread of infection to other flocks, and thereby avoiding potential losses and risks for animals and humans that would probably have occurred with use of traditional methods [63]. An alternative approach to animal-movement pattern monitoring is automatic image acquisition and analysis. Eyenamic^TM^ software has been used to calculate birds’ activity levels by processing calibrated recorded video images. The differences in pixel intensity values with respect to those of the previous image enable calculation of an activity index. This system has been used to assess the relationship between automatic gait evaluation with gait scores obtained by human experts and to develop an automatic activity-index tool capable of detecting leg problems [33,40]. Another approach, optical flow analysis (OF), developed for applications such as traffic flows, movement of glaciers, or cell and sperm motility, has also been applied in the analysis of movement in confined broilers [15,33]. OF may provide a practical approach for the assessment of movement-associated welfare issues in commercial poultry through the automatic and continuous assessment of moving images containing hundreds of individuals [64]. Recent studies have indicated that OF technology can even be useful in detecting Campylobacter-infected flocks. Colles et al. have shown that flocks likely to become positive for Campylobacter can be identified in the first 7–10 days of life, and are characterized by a lower mean flow rate and consistently higher kurtosis than observed in non-infected flocks [65]. If positive results continue to be supported by research, these technologies may greatly influence poultry management, because they benefit animals, producers, and consumers by reducing economic losses and improving food safety. Furthermore, these methods are non-invasive and relatively easy to apply in large flocks. It is probably only a matter of time before OF and other technologies are commonly applied to commercial laying hens or other poultry species. Among imaging techniques, infrared imaging can determine the surface temperatures of objects and create image maps with colors representing different temperatures [66]. Heat stress is detrimental to poultry health, and body temperature is indicative of physiological abnormalities that can lead to elevated rates of mortality. Infrared thermal imaging can be used to detect chicken temperature after changes in diet, poultry house environments, and stress levels [1]. Near-infrared spectroscopy has been applied in the assessment of the barn thermal environment, and in compliance regarding comfort zones and insulation [67]. Other aspects of meat production have benefitted from this technology, such as the non-destructive detection and grading of wooden breast syndrome in chicken breast fillets [68]. The above examples describe only a few of the plethora of automatic data generation systems that are already available or are under development for the poultry industry. Further benefits are, and will be, associated with the common implementation of mobile apps dedicated to welfare, health, and productive performance assessment, because they provide easy and user-friendly access to substantial computational power and connectivity, and enable extremely effective geolocation. Therefore, they have clear applications in monitoring and reconstructing the movements of employees, trucks, and other fomites, as well as in evaluating whether established flows and biosecurity measures are being followed [9,69,70].

**Figure 1:**
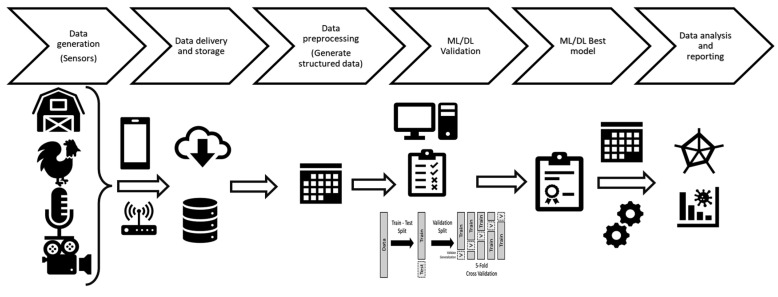
Potential poultry-farm-generated data flow, from collection to output generation.

Although not exhaustive, the reported overview of data collection methods and sensors demonstrate the breadth of fields that can be investigated using different technologies, ranging from management efficacy improvement and assessment to animals’ welfare and health monitoring, biosecurity implementation, early-stage disease detection in animals, etc. Nevertheless, the amount and variability of generated data can be dispersive and hamper their interpretability. Therefore, proper data organization, analysis, and reporting are mandatory to produce an effective output and fully benefit from the obtained information (Figure 1).

### 2.2. Data Management: Computational Approaches, Storage and Sharing

As new sensors and technologies become incorporated into poultry farming operations, larger amounts of data will be generated. Such development must be paired with adequate infrastructure for collecting, interpreting, and applying all this information. Local resources are typically insufficient for such purposes, and connectivity in a broader sense is critically important. The internet of things (IoT) is leading to massive changes in how humans live and work. The IoT infrastructure consists of several components, including hardware to collect data from the environment; connectivity to transmit data; software to store, analyze and process data; and an interface to allow users to interact with the IoT platform [71]. The implementation of IoT technologies in poultry production will consist of a variety of internet-connected smart devices that enable enhanced device communication, thereby leading to automation of operations, and allowing humans to focus on monitoring farms and act on processes requiring higher levels of intelligence [72]. The main advantage that IoT provides for the poultry industry is the capabilitiy for communication between sensors and equipment that are used on the farm; storage of information in remote or cloud datasets; analysis of data with algorithms requiring intensive computational resources; and provision of an automatic response action or feedback to farmers [72]. The need for more complex data processing and analysis approaches is a key feature of big data. Basic and traditional statistical models, based primarily on variants and extensions of linear regression models, are typically unsuitable for large datasets including several parameters, and for modeling the large variability and complexity of biological phenomena and productive processes. The application of ML and deep learning (DL) algorithms is thus becoming increasingly common [11,73,74,75]. ML refers to computer systems and algorithms that can learn and adapt automatically from experience (i.e., from data) without being explicitly programmed. ML typically requires the input data to be pre-processed to make them more amenable to processing by these methods (so-called “feature engineering”). DL, in contrast, can be viewed as a further extension that completely automates this step. The use of a complex structure of algorithms such as artificial neural networks inspired by the human brain enables the processing of unstructured data. These advances have greatly simplified ML workflows, and sophisticated multistage pipelines have often been replaced by a single simple end-to-end DL model [76,77]. In recent years, these methods have found many applications in all sectors of society and have demonstrated excellent categorization and prediction capabilities. However, because of the complexity of the methods and the data that they address, the interpretability is limited or absent [76]. The methods behave in a manner similar to “black boxes”, to which inputs are provided, and from which outputs are received; therefore, the underlying causes, intermediate processing, mathematical models, and relevance of the different variables involved are obscure [75]. This aspect differentiates these approaches from traditional statistical ones, whose mathematical formulations are well-known and operator-defined, and are based primarily on causal association, either known or hypothesized. Consequently, considerable mistrust in ML and DL has arisen among non-experts in the field. A brief explanation of the key principles of ML and DL development and validation is thus warranted. In most instances, ML and DL are used to predict a quantitative or categorical outcome. For this purpose, the methods learn (are “trained”) from a dataset of records with known features and outcomes of interest. During the training, the method parameters are automatically optimized to maximize predictive performance (i.e., minimize errors). Nevertheless, the effectiveness of the developed tool in predicting future data is not ensured. That is, the tool could be too specific for the training dataset, and the prediction could be inaccurate for external data. Therefore, an additional check must be performed on a test dataset, i.e., a dataset with the same features as the training dataset (and comparable with the datasets that will be provided thereafter, during application of the routine method) and with known outcomes, whose records were not used in the training step. In this way, an objective and empirical evaluation of the performance and generalizability of the ML or DL approach can be demonstrated, thus ensuring its applicability to future data. Therefore, although the process might appear obscure, its reliability can be considered to be even higher than that of traditional methods, being validated on the basis of empirical demonstrations rather than mathematical assumptions. The outcome of this process is an automatic response or an easily understandable and effective warning/reporting system for farmers or other workers. Typically, farmers address diseases in their animals by taking no action, proactively using veterinary physicians, using a mix of antibiotics, or, in many cases, following a combination of these three approaches [10]. Modern technologies such as sensors, big data, AI, and ML present new possibilities for farmers. Instead of reacting to diseases after they become evident, farmers can continuously monitor key animal health parameters, such as movement, air quality, and consumption of food and fluids. By collecting these data and using advanced AI and ML algorithms to predict deviations or abnormalities, farmers can now identify, predict, and prevent disease outbreaks, even before large-scale outbreaks occur. That is, sensors, instead of humans, can perform continuous monitoring of animal health [63,78]. The first advantage of this system is that it enables fewer farmers to care for many more animals, thereby decreasing production costs [10]. Second, this system can notify farmers about the possibility of a disease, even during pre-clinical stages, thus helping farmers take timely action to prevent catastrophic losses [10,63,79].

## 3. Molecular Epidemiology of Pathogens

**Figure 2:**
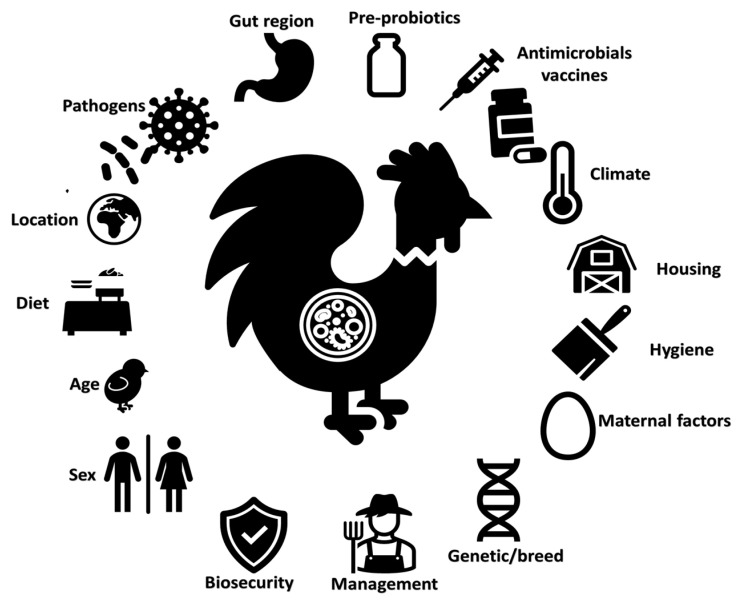
Overview of factors that may affect the intestinal microbiota composition in poultry.

Another field in which data generation has greatly increased is the production of genetic sequences. The development and continuing improvement of next-generation sequencing (NGS) and third generation sequencing (TGS) has represented a true revolution in perspective [80,81,82]. Modern sequencing platforms can generate an amount of genetic data for each sample which was unthinkable only a few years ago and cannot be interpreted by the operator without the assistance of appropriate analysis software and adequate computational power. At the same time, the amount and variability of analyzed specimens are exponentially increasing over years, as well as the parallel increases in the availability of the sequence in freely available databases. As a result, this field too can rightfully be included in the sphere of big data analytics. Because the increase in sequence-data yield is faster than the increase in computer processing power, NGS has forced researchers to rethink more than their software. Aspects including storage, processing power, and data output are being retrofitted or redesigned to meet the demands of ever-faster sequencing machines [83]. This improvement in sequencing capability has rapidly increased knowledge regarding the genetics of hosts, host microbiota, and pathogens; gene expression; metabolic patterns; and epidemiology, among other aspects. Because a comprehensive discussion of all these topics would not be feasible herein, this review focuses on the use of molecular and big data in the study of microbiome and pathogen features, epidemiology, and evolution, and their applications in the poultry sector. In past decades, Sanger sequencing has been used to obtain relatively short (~1 kb) sequences of the genes of target microorganisms. Although this approach is valuable and is still commonly applied for routine diagnostic and research purposes, it has some limitations, including the short length of the obtained sequences (thus decreasing the resolution of strain comparisons), the need for a priori knowledge of the target sequence to design specific primers and probes, and challenges in the investigation of within-sample microbial diversity and the presence of subpopulations, both of which are fundamental for microbiota or rapidly evolving RNA or ssDNA viruses’ characterization [84,85,86]. NGS and TGS, despite the peculiarities associated with each method, have largely solved most of these problems. The massive sequencing capability allows the complete genomes of viruses and bacteria to be easily obtained, thereby increasing characterization capabilities and the resolution of epidemiological studies to unprecedented levels, as well as leading to the establishment of robust epidemiological links among animals [87] or humans (e.g., zoonotic foodborne pathogens) [88]. Moreover, the ability to perform primer-independent sequencing enables whole-genome sequencing of unknown pathogens or highly divergent variants of known pathogens, in which primer mismatches can severely affect assay sensitivity. Bali et al. have recently used a combination of random amplification and NGS to identify and characterize a new lineage of IBV in sub-Saharan Africa [89]. A new chaphamaparvovirus has also been characterized and associated with several outbreaks of hepatitis in flocks of young pheasants in France—a pathology first described 50 years ago, whose etiology had remained obscure [90]. The implications of such advancements in disease monitoring and specific control strategies implementation are clear. A similar approach has been implemented in metagenomic studies (i.e., the study of the structure and function of entire nucleotide sequences isolated and analyzed from all organisms (typically microorganisms) in bulk samples), thus enabling the study of the composition of the host microbiome and the characterization of expressed genes [91]. The metabolic pathways, and the presence of virulence and antimicrobial resistance genes, can thus be investigated in a quali-quantitative manner [92]. Because of the extensive links among microbiota, microbiota metabolism, host physiology, productivity, and health, the investigation of microbial community changes after various treatments (e.g., diets, housing conditions, or administration of antimicrobial agents and probiotics, etc; Figure 2) can enable direct interventions that maximize animal welfare and farm profitability [93,94,95].

Another benefit of parallel sequencing capabilities is the ability to “read” the same region of the genome hundreds or thousands of times. Because each sequence originates from a single DNA/RNA molecule, NGS and TGS enable the study of the presence, frequency, and structure of microorganism subpopulations. This information is of particular relevance for RNA and ssDNA viruses, whose evolutive potential is so high that they can be depicted as a within-host swarm of variants emerging from a main consensus population [96,97]. Although most variants have limited fitness, some might be advantageous and consequently be selected and spread on a broader epidemiological scale [97,98]. Similar phenomena have also been observed with live attenuated vaccines: subpopulations have frequently been identified, and different vaccines or production batches can exhibit variable heterogenicity or presence in specific subpopulations [86,99,100]. This finding has several implications because the population structure of vaccines has been reported to affect innate immune responses, antibody avidity, and protection. For example, IBV vaccine heterogeneity is associated with a differential host response [101,102]. A comparison between a commercial Arkansas Delmarva Poultry Industry (ArkDPI)-type vaccine and a more homogeneous population of the same vaccine obtained through adaptation to chicken embryo kidney cells (CEK-ArkDPI) has indicated that the more heterogeneous commercial ArkDPI was more efficient in decreasing viral loads in challenged chickens, although the antibody levels and antibody avidity to the Ark-type S1 protein were greater in CEK-ArkDPI-vaccinated chickens. The virus population showing increased diversity (commercial ArkDPI) achieved higher concentrations of IBV RNA in the trachea than did the more homogeneous CEK-ArkDPI population, thus probably leading to higher mRNA expression of genes associated with innate immune responses [102].

Moreover, the occurrence of reversion-to-virulence phenomena might be favored because of the persistence of partially attenuated variants or sub-populations pre-adapted for in vivo replication, or a combination of both [86,103]. The occurrence of within-vaccine variants and/or inter-batch variability might also complicate the differentiation between vaccine and field strains through genomic sequence analysis by preventing the definition of a certain reference vaccine sequence [100]. After sequences have been generated through Sanger sequencing or NGS, they can be analyzed in many different ways. Although traditional sequence comparison and phylogenetic analysis remain extremely useful for strain classification (e.g., genotyping, field-vaccine strain differentiation, etc.), other approaches are able to include large amounts of metadata to be modeled in the analysis together with the genetic data. Phylodynamic analysis allows for the investigation of the effects of epidemiological, evolutive, and immunological forces on phylogeny. The central premise of this discipline is that epidemic processes, such as viral population growth and subdivision, leave a measurable imprint on the genomes of viruses over the course of years, months, or even individual days, one which can be investigated with appropriate mathematical models [104]. Molecular clocks have been used to infer viral origin or introduction in a country, thereby enabling, for example, assessment of the effectiveness of surveillance and early-detection systems [105,106]. Pathogen population dynamics and history (i.e., the variation in population size over time) can be reconstructed through analysis of viral phylogeny, thus avoiding, or at least limiting, the biasing effect of variations in diagnostic and sequencing activity among countries or periods, and allowing for a more objective evaluation of factors contributing to either the success of viral spread or the efficacy of implemented control measures. A strong effect of vaccination strategies against IBV has been demonstrated in Italy against the circulating QX (GI-19) lineage. Interestingly, the number of clinical outbreaks was higher when a heterologous Mass + 793B or homologous Mass + QX was applied, than with only Mass administration [107]. However, when the viral population size was reconstructed through a phylodynamic approach, an opposite effect was observed. This difference might be explained by lag phases after managerial changes, progressive increases in infectious pressure, and disease emergence. Nevertheless, the evaluation of clinical consequences would notably have led to an erroneous conclusion regarding the detrimental effects of vaccine application [107]. By linking strain genetic data and knowledge regarding geographical locations, phylogeographic reconstruction can be performed, particularly for rapidly evolving viruses [104,108]. For these rapidly evolving pathogens, sequence evolution occurs simultaneously with geographic dispersal, and the geographic location is treated as an inherited property of the virus. The aim is to estimate the ancestral locations in a phylogenetic tree (i.e., reconstruct viral migration over time), according to the observed locations of viral sequences represented by the tips of the tree. Interestingly, these approaches are not necessarily limited to spatial traits, but can be used to study how viruses spread in different hosts, phenotypes, poultry companies, etc., over time [109]. Many examples of poultry infections are available to help predict the emergence of infectious diseases by identifying key reservoir species, and the geographic areas from which new infections are likely to arise and spread [104,110,111]. More recent approaches have included formal statistical tests in the phylogeographic framework to evaluate location features (e.g., geographic distance, animal population size, trades, sharing of services, or viral features) significantly determining the likelihood of viral dispersion [112]. Similarly, the effects of the features of the local landscape (e.g., altitude, animal population density, road density, or climate) can be integrated as variables enhancing or hindering viral dispersal [113,114,115]. Poultry density has been demonstrated as the only factor significantly affecting IBV dispersal in Italy [109], whereas road density has been proven fundamental for other pathogens of veterinary interest, such as the porcine reproductive and respiratory syndrome virus [116], thus demonstrating how viral features and farming system organization can differentially affect infection epidemiology. Finally, the strengths and features of selective forces acting on and shaping pathogen evolution can be inferred through the analysis of gene sequences and comparison between populations of pathogens subjected to different conditions (e.g., different locations or time periods) or control strategies (i.e., vaccination) [117,118,119,120]. A stronger selective pressure acting on viruses circulating in environments where homologous vaccination instead of heterologous vaccination is applied has been demonstrated through such a bioinformatics approach [121]. New methods and extensions are continually being developed to integrate increasing heterogeneous metadata in a common framework and account for the features of poultry farming. Simultaneously, the increasing submission of sequences and associated data to public databases allows the implementation of analysis with thousands or tens of thousands of records, thus leading to increasingly accurate models for the understanding and prediction of pathogen epidemiology.

## 4. Critical Points and Challenges

Despite the clear advantages of big data analytics, sensor platforms and ML application, some drawbacks and sensitive issues cannot be ignored.

First, the ownership, use, and privacy of the data constitute problems [122,123]. Vast amounts of data from technology products and services are stored on remote cloud servers and are often monetized for commercial benefits. Some large corporations collect, use, and even sell farmers’ data. The rising tensions between farmers and service providers regarding data misuse is a considerable threat that may prevent or limit technology applications [72]. Moreover, in some instances, technology cannot be used effectively. In some cases, farmers may be reluctant or unable to use the latest technology in their farms because of various economic, social, environmental, physical, and situational constraints [124,125]. Whether and how big data analysis applications in farming will affect and magnify social inequity is a topic that must not be overlooked. Finally, companies are being criticized for selling premature technologies to farmers without sufficient trials or evidence. Many people strongly believe that technology companies are using farmers to validate their products and services, thus de-risking themselves at the expense of the final users. Because many of these technologies remain at their nascent stages, any mistakes could result in costly damages for farmers and a loss of confidence [10]. Another sensitive topic relates to infectious disease sequences and metadata sharing. Most farmers and poultry companies are reluctant to disclose such information and are afraid of potential misuses that might cause commercial disadvantages or legal disputes [126]. Simultaneously, pharmaceutical companies and diagnostic laboratories tend not to distribute data, and to take advantage of their exclusive knowledge, although the ownership of data obtained during routine diagnostic activity is a sensitive topic that should be further considered. Similarly, and even more regrettably, public institutions and research centers do not regularly share the acquired data, in order to protect their imprimatur or knowledge-advantage in specific topics. This reluctance can have severe consequences, worsening the potential bias in data distribution (e.g., only favorable information, information originating from a subset of companies with a proactive attitude, or outdated information might be shared) or hindering the proper ML/DL algorithms’ validation through cross-validation and test dataset evaluation because of limited/partial records availability. A standardized approach to data collection should be pursued as far as possible to evaluate and account for inter-farm/-area/-country variability and improve the generalizability of the developed algorithms.

Sharing of data and sequences with adequate associated metadata is key to the successful interpretation of microorganisms’ behavior, infectious disease epidemiology, and the efficacy of control strategies. Therefore, data sharing should be adequately encouraged, ensuring the necessary degree of anonymity without the loss of associated information, and generating easily-understandable reports that could benefit the farmers and companies providing the data, thus indirectly benefitting all of society, and animal and human health.

## 5. Conclusions

Although several unknowns, limitations, and open-ended questions remain—and the human-artificial intelligence collaborations in the livestock sector can be frightening, because they require farmers and employees in the sector to accommodate new roles, challenges and competencies—the overall benefits appear overwhelming. Despite recent developments, a lack of both standardization and willingness to participate in global collection and sharing of production and molecular data continue to persist. However, as more farms and companies become connected to technology, AI and sensing technologies are expected to play a greater role in identifying patterns and solutions to pressing problems in modern animal farming, thus providing a remarkably productive and commercial advantage. Moreover, the combination of diverse sources and types of data, including farm-generated and trade data, as well as data on climate and human activities, will also become fundamental for the development of predictive models that can anticipate, rather than merely detect, disease occurrence. The increasing availability of sensors, infrastructures, and tools for big data collection, storage, sharing, and analysis, together with the use of open standards and the integration with pathogen molecular epidemiology, have the potential to address the challenge of producing higher-quality, more healthful food on a larger scale in more sustainable ways, thus protecting physical ecosystems, preserving natural resources, and improving animal and human welfare and health.

## Figures and Tables

**Table 1:** Examples of sensors used to evaluate and detect alteration in different fields of poultry farming.

Field	Topic	Sensor	Reference
Infectious disease	Avian influenza	Wearable sensor	[18,19,20,21,22,23,24,25]
		Imaging	
		Sound analysis	
		Thermal images	
	Clostridium perfringens	Sound analysis	[26]
	Coccidiosis	Volatile organic compounds	[27,28,29]
		Imaging	
	Infectious bronchitis	Sound analysis	[23,24,25]
	Newcastle disease	Sound analysis	[23,30,31,32,33]
		Imaging	
Welfare and health	Distress	Thermal Imaging	[34,35,36,37]
		Imaging	
	Footpad dermatitis	Imaging	[15,38]
	Gait score and lameness	Imaging	[35,39,40]
	Management and equipment malfunctioning	Imaging	[33,41]
	Thermal comfort	Sound analysis	[37,42]
Production	Broiler performances	Feed nutritional composition	[43]
	Chicken embryo sex assessment	Raman Spectroscopy	[44,45,46]
	Egg production	Multiple Environmental Sensors	[47,48]
	Embryo monitoring	Thermal Images	[49,50]
	Live weight of broilers	Imaging	[51,52]
	Poultry house environmental monitoring	Multiple Environmental Sensors	[53,54,55,56]
	Precision feeding systems	Weight Sensor	[57,58,59]
		Thermal Images	

## Data Availability

Not applicable.

## References

[1] Astill J., Dara R.A., Fraser E.D.G., Roberts B., Sharif S. (2020). Smart Poultry Management: Smart Sensors, Big Data, and the Internet of Things. Comput. Electron. Agric..

[2] Farrell D. (2013). The Role of Poultry in Human Nutrition. Poultry Development Review.

[3] Aklilu H.A., Almekinders C.J.M., Udo H.M.J., Van Der Zijpp A.J. (2007). Village Poultry Consumption and Marketing in Relation to Gender, Religious Festivals and Market Access. Trop. Anim. Health Prod..

[4] Solomon D. (2008). Ethiopia: Poultry Sector Country Review.

[5] Pearson D., Gorman J., Aspinall R. (2022). Multiple Roles for Landscape Ecology in Future Farming Systems: An Editorial Overview. Land.

[6] Cravero A., Sepúlveda S. (2021). Use and Adaptations of Machine Learning in Big Data—Applications in Real Cases in Agriculture. Electronics.

[7] Ouyang Z., Sargeant J., Thomas A., Wycherley K., Ma R., Esmaeilbeigi R., Versluis A., Stacey D., Stone E., Poljak Z. (2019). A Scoping Review of “Big Data”, “Informatics”, and “bioinformatics” in the Animal Health and Veterinary Medical Literature. Anim. Health Res. Rev..

[8] Sharma V., Tripathi A.K., Mittal H. (2022). Technological Revolutions in Smart Farming: Current Trends, Challenges & Future Directions. Comput. Electron. Agric..

[9] Bolfe É.L., de Jorge L.A.C., Sanches I.D., Júnior A.L., da Costa C.C., de Castro Victoria D., Inamasu R.Y., Grego C.R., Ferreira V.R., Ramirez A.R. (2020). Precision and Digital Agriculture: Adoption of Technologies and Perception of Brazilian Farmers. Agriculture.

[10] Neethirajan S. (2020). The Role of Sensors, Big Data and Machine Learning in Modern Animal Farming. Sens. Biosensing Res..

[11] Liakos K.G., Busato P., Moshou D., Pearson S., Bochtis D. (2018). Machine Learning in Agriculture: A Review. Sensors.

[12] Diebold F.X. (2012). On the Origin(s) and Development of the Term “Big Data”. SSRN Electron. J..

[13] Shafer T. The 42 V’s of Big Data and Data Science—KDnuggets. https://www.kdnuggets.com/2017/04/42-vs-big-data-data-science.html.

[14] Wathes C.M., Kristensen H.H., Aerts J.M., Berckmans D. (2008). Is Precision Livestock Farming an Engineer’s Daydream or Nightmare, an Animal’s Friend or Foe, and a Farmer’s Panacea or Pitfall?. Comput. Electron. Agric..

[15] Sassi N.B., Averós X., Estevez I. (2016). Technology and Poultry Welfare. Animals.

[16] Dawkins M.S., Donnelly C.A., Jones T.A. (2004). Chicken Welfare Is Influenced More by Housing Conditions than by Stocking Density. Nature.

[17] Meluzzi A., Sirri F. (2016). Welfare of Broiler Chickens. Ital. J. Anim. Sci..

[18] Okada H., Itoh T., Suzuki K., Tsukamoto K. Wireless Sensor System for Detection of Avian Influenza Outbreak Farms at an Early Stage. Proceedings of the SENSORS.

[19] Cuan K., Zhang T., Huang J., Fang C., Guan Y. (2020). Detection of Avian Influenza-Infected Chickens Based on a Chicken Sound Convolutional Neural Network. Comput. Electron. Agric..

[20] Huang J., Wang W., Zhang T. (2019). Method for Detecting Avian Influenza Disease of Chickens Based on Sound Analysis. Biosyst. Eng..

[21] Zhuang X., Bi M., Guo J., Wu S., Zhang T. (2018). Development of an Early Warning Algorithm to Detect Sick Broilers. Comput. Electron. Agric..

[22] Banakar A., Sadeghi M., Shushtari A. (2016). An Intelligent Device for Diagnosing Avian Diseases: Newcastle, Infectious Bronchitis, Avian Influenza. Comput. Electron. Agric..

[23] Mahdavian A., Minaei S., Marchetto P.M., Almasganj F., Rahimi S., Yang C. (2021). Acoustic Features of Vocalization Signal in Poultry Health Monitoring. Appl. Acoust..

[24] Rizwan M., Carroll B.T., Anderson D.V., Daley W., Harbert S., Britton D.F., Jackwood M.W. Identifying Rale Sounds in Chickens Using Audio Signals for Early Disease Detection in Poultry. Proceedings of the 2016 IEEE Global Conference on Signal and Information Processing (GlobalSIP).

[25] Carroll B.T., Anderson D.V., Daley W., Harbert S., Britton D.F., Jackwood M.W. Detecting Symptoms of Diseases in Poultry through Audio Signal Processing. Proceedings of the 2014 IEEE Global Conference on Signal and Information Processing (GlobalSIP).

[26] Sadeghi M., Banakar A., Khazaee M., Soleimani M.R. (2015). An Intelligent Procedure for the Detection and Classification of Chickens Infected by Clostridium Perfringens Based on Their Vocalization. Rev. Bras. Cienc. Avic..

[27] Mbelwa H., Machuve D., Mbelwa J. (2021). Deep Convolutional Neural Network for Chicken Diseases Detection. Int. J. Adv. Comput. Sci. Appl..

[28] Borgonovo F., Ferrante V., Grilli G., Pascuzzo R., Vantini S., Guarino M. (2020). A Data-Driven Prediction Method for an Early Warning of Coccidiosis in Intensive Livestock Systems: A Preliminary Study. Animals.

[29] Grilli G., Borgonovo F., Tullo E., Fontana I., Guarino M., Ferrante V. (2018). A Pilot Study to Detect Coccidiosis in Poultry Farms at Early Stage from Air Analysis. Biosyst. Eng..

[30] Cuan K., Zhang T., Li Z., Huang J., Ding Y., Fang C. (2022). Automatic Newcastle Disease Detection Using Sound Technology and Deep Learning Method. Comput. Electron. Agric..

[31] Okinda C., Lu M., Liu L., Nyalala I., Muneri C., Wang J., Zhang H., Shen M. (2019). A Machine Vision System for Early Detection and Prediction of Sick Birds: A Broiler Chicken Model. Biosyst. Eng..

[32] Carpentier L., Vranken E., Berckmans D., Paeshuyse J., Norton T. (2019). Development of Sound-Based Poultry Health Monitoring Tool for Automated Sneeze Detection. Comput. Electron. Agric..

[33] Kashiha M., Pluk A., Bahr C., Vranken E., Berckmans D. (2013). Development of an Early Warning System for a Broiler House Using Computer Vision. Biosyst. Eng..

[34] Ren Y., Johnson M.T., Clemins P.J., Darre M., Glaeser S.S., Osiejuk T.S., Out-Nyarko E. (2009). A Framework for Bioacoustic Vocalization Analysis Using Hidden Markov Models. Algorithms.

[35] Dawkins M.S., Lee H.J., Waitt C.D., Roberts S.J. (2009). Optical Flow Patterns in Broiler Chicken Flocks as Automated Measures of Behaviour and Gait. Appl. Anim. Behav. Sci..

[36] Edgar J.L., Paul E.S., Nicol C.J. (2009). Thermal Imaging as a Non-Invasive Tool to Assess Mild Distress in Chickens. World Poultry Science Association (WPSA), Proceedings of the 8th European Symposium on Poultry Welfare, Cervia, Italy, 18–22 May 2009.

[37] Mollo M.N., Vendrametto O., Okano M.T. (2010). Precision Livestock Tools to Improve Products and Processes in Broiler Production: A Review. Rev. Bras. Cienc. Avic..

[38] Hepworth P.J., Nefedov A.V., Muchnik I.B., Morgan K.L. (2012). Broiler Chickens Can Benefit from Machine Learning: Support Vector Machine Analysis of Observational Epidemiological Data. J. R. Soc. Interface.

[39] Aydin A., Cangar O., Ozcan S.E., Bahr C., Berckmans D. (2010). Application of a Fully Automatic Analysis Tool to Assess the Activity of Broiler Chickens with Different Gait Scores. Comput. Electron. Agric..

[40] Silvera A.M., Knowles T.G., Butterworth A., Berckmans D., Vranken E., Blokhuis H.J. (2017). Lameness Assessment with Automatic Monitoring of Activity in Commercial Broiler Flocks. Poult. Sci..

[41] Cordeiro M.B., Tinôco I.F.F., De Mesquita Filho R.M., De Sousa F.C. (2011). Digital Image Analysis for Young Chicken’s Behavior Evaluation. Eng. Agric..

[42] De Moura D.J., Nääs I.D.A., Alves E.C.D.S., De Carvalho T.M.R., Do Vale M.M., De Lima K.A.O. (2008). Noise Analysis to Evaluate Chick Thermal Comfort. Sci. Agric..

[43] Faridi A., Sakomura N.K., Golian A., Marcato S.M. (2012). Predicting Body and Carcass Characteristics of 2 Broiler Chicken Strains Using Support Vector Regression and Neural Network Models. Poult. Sci..

[44] Krautwald-Junghanns M.E., Cramer K., Fischer B., Förster A., Galli R., Kremer F., Mapesa E.U., Meissner S., Preisinger R., Preusse G. (2018). Current Approaches to Avoid the Culling of Day-Old Male Chicks in the Layer Industry, with Special Reference to Spectroscopic Methods. Poult. Sci..

[45] Galli R., Preusse G., Schnabel C., Bartels T., Cramer K., Krautwald-Junghanns M.E., Koch E., Steiner G. (2018). Sexing of Chicken Eggs by Fluorescence and Raman Spectroscopy through the Shell Membrane. PLoS ONE.

[46] Galli R., Preusse G., Uckermann O., Bartels T., Krautwald-Junghanns M.E., Koch E., Steiner G. (2016). In Ovo Sexing of Domestic Chicken Eggs by Raman Spectroscopy. Anal. Chem..

[47] Bumanis N., Kviesis A., Tjukova A., Arhipova I., Paura L., Vitols G. (2023). Smart Poultry Management Platform with Egg Production Forecast Capabilities. Procedia Comput. Sci..

[48] Morales I.R., Cebrián D.R., Fernandez-Blanco E., Sierra A.P. (2016). Early Warning in Egg Production Curves from Commercial Hens: A SVM Approach. Comput. Electron. Agric..

[49] Exadaktylos V., Silva M., Berckmans D. (2011). Real-Time Analysis of Chicken Embryo Sounds to Monitor Different Incubation Stages. Comput. Electron. Agric..

[50] Shinder D., Rusal M., Giloh M., Yahav S. (2009). Effect of Repetitive Acute Cold Exposures during the Last Phase of Broiler Embryogenesis on Cold Resistance through the Life Span. Poult. Sci..

[51] Mollah M.B.R., Hasan M.A., Salam M.A., Ali M.A. (2010). Digital Image Analysis to Estimate the Live Weight of Broiler. Comput. Electron. Agric..

[52] Johansen S.V., Bendtsen J.D., Jensen R.M., Mogensen J. (2019). Broiler Weight Forecasting Using Dynamic Neural Network Models with Input Variable Selection. Comput. Electron. Agric..

[53] Jackman P., Ward S., Brennan L., Corkery G., McCarthy U. (2015). Application of Wireless Technologies to Forward Predict Crop Yields in the Poultry Production Chain. Agric. Eng. Int. CIGR J..

[54] Flocking to Digital: Re-Imagining the Future of Poultry through Innovation. https://www.linkedin.com/pulse/how-technology-transforming-poultry-industry-aidan-connolly-7k-.

[55] Bustamante E., Guijarro E., García-Diego F.J., Balasch S., Hospitaler A., Torres A.G. (2012). Multisensor System for Isotemporal Measurements to Assess Indoor Climatic Conditions in Poultry Farms. Sensors.

[56] David B., Mejdell C., Michel V., Lund V., Moe R.O. (2015). Air Quality in Alternative Housing Systems May Have an Impact on Laying Hen Welfare. Part II—Ammonia. Animals.

[57] Zuidhof M.J., Fedorak M.V., Ouellette C.A., Wenger I.I. (2017). Precision Feeding: Innovative Management of Broiler Breeder Feed Intake and Flock Uniformity. Poult. Sci..

[58] Ferreira V., Francisco N., Belloni M., Aguirre G., Caldara F., Nääs I., Garcia R., Almeida Paz I., Polycarpo G. (2011). Infrared Thermography Applied to the Evaluation of Metabolic Heat Loss of Chicks Fed with Different Energy Densities. Rev. Bras. Cienc. Avic..

[59] Zuidhof M.J. (2018). Lifetime Productivity of Conventionally and Precision-Fed Broiler Breeders. Poult. Sci..

[60] Zimmerman P.H., Koene P., Van Hooff J.A.R.A.M. (2000). The Vocal Expression of Feeding Motivation and Frustration in the Domestic Laying Hen, Gallus Gallus Domesticus. Appl. Anim. Behav. Sci..

[61] Pereira E., de Nääs I.A., Ivale A.H., Garcia R.G., da Lima N.D.S., Pereira D.F. (2023). Energy Assessment from Broiler Chicks’ Vocalization Might Help Improve Welfare and Production. Animals.

[62] Bright A. (2008). Vocalisations and Acoustic Parameters of Flock Noise from Feather Pecking and Non-Feather Pecking Laying Flocks. Br. Poult. Sci..

[63] Astill J., Fraser E., Dara R., Sharif S. (2018). Detecting and Predicting Emerging Disease in Poultry with the Implementation of New Technologies and Big Data: A Focus on Avian Influenza Virus. Front. Vet. Sci..

[64] Roberts S.J., Cain R., Dawkins M.S. (2012). Prediction of Welfare Outcomes for Broiler Chickens Using Bayesian Regression on Continuous Optical Flow Data. J. R. Soc. Interface.

[65] Colles F.M., Cain R.J., Nickson T., Smith A.L., Roberts S.J., Maiden M.C.J., Lunn D., Dawkins M.S. (2016). Monitoring Chicken Flock Behaviour Provides Early Warning of Infection by Human Pathogen Campylobacter. Proc. R. Soc. B Biol. Sci..

[66] Nääs I.A., Garcia R.G., Caldara F.R. (2020). Infrared Thermal Image for Assessing Animal Health and Welfare. J. Anim. Behav. Biometeorol..

[67] Corrand L., Brelaz M., Guerin J.L. (2010). The Use of Infrared Thermography for Evaluating the Environment in Poultry Buildings. TeMA Tech. Et Marchés Avic..

[68] Wold J.P., Veiseth-Kent E., Høst V., Løvland A. (2017). Rapid On-Line Detection and Grading of Wooden Breast Myopathy in Chicken Fillets by near-Infrared Spectroscopy. PLoS ONE.

[69] Daum T., Buchwald H., Gerlicher A., Birner R. (2018). Smartphone Apps as a New Method to Collect Data on Smallholder Farming Systems in the Digital Age: A Case Study from Zambia. Comput. Electron. Agric..

[70] Pongnumkul S., Chaovalit P., Surasvadi N. (2015). Applications of Smartphone-Based Sensors in Agriculture: A Systematic Review of Research. J. Sens..

[71] Madakam S., Ramaswamy R., Tripathi S. (2015). Internet of Things (IoT): A Literature Review. J. Comput. Commun..

[72] Wolfert S., Ge L., Verdouw C., Bogaardt M.J. (2017). Big Data in Smart Farming—A Review. Agric. Syst..

[73] Ojo R.O., Ajayi A.O., Owolabi H.A., Oyedele L.O., Akanbi L.A. (2022). Internet of Things and Machine Learning Techniques in Poultry Health and Welfare Management: A Systematic Literature Review. Comput. Electron. Agric..

[74] Cravero A., Pardo S., Sepúlveda S., Muñoz L. (2022). Challenges to Use Machine Learning in Agricultural Big Data: A Systematic Literature Review. Agronomy.

[75] Jordan M.I., Mitchell T.M. (2015). Machine Learning: Trends, Perspectives, and Prospects. Science.

[76] Lecun Y., Bengio Y., Hinton G. (2015). Deep Learning. Nature.

[77] Ketkar N., Santana E. (2017). Deep Learning with Python.

[78] VanderWaal K., Morrison R.B., Neuhauser C., Vilalta C., Perez A.M. (2017). Translating Big Data into Smart Data for Veterinary Epidemiology. Front. Vet. Sci..

[79] Morota G., Ventura R.V., Silva F.F., Koyama M., Fernando S.C. (2018). Big Data Analytics and Precision Animal Agriculture Symposium: Machine Learning and Data Mining Advance Predictive Big Data Analysis in Precision Animal Agriculture. J. Anim. Sci..

[80] van Dijk E.L., Jaszczyszyn Y., Naquin D., Thermes C. (2018). The Third Revolution in Sequencing Technology. Trends Genet..

[81] Hu T., Chitnis N., Monos D., Dinh A. (2021). Next-Generation Sequencing Technologies: An Overview. Hum. Immunol..

[82] Muzzey D., Evans E.A., Lieber C. (2015). Understanding the Basics of NGS: From Mechanism to Variant Calling. Curr. Genet. Med. Rep..

[83] Baker M. (2010). Next-Generation Sequencing: Adjusting to Data Overload. Nat. Methods.

[84] van Rijn-Klink A., De Vries J.J.C., Claas E.C.J. (2021). Next-Generation Sequencing in Clinical Virology. Application and Integration of Omics-Powered Diagnostics in Clinical and Public Health Microbiology.

[85] Deurenberg R.H., Bathoorn E., Chlebowicz M.A., Couto N., Ferdous M., García-Cobos S., Kooistra-Smid A.M.D., Raangs E.C., Rosema S., Veloo A.C.M. (2017). Application of next Generation Sequencing in Clinical Microbiology and Infection Prevention. J. Biotechnol..

[86] Franzo G., Naylor C.J., Drigo M., Croville G., Ducatez M.F., Catelli E., Laconi A., Cecchinato M. (2015). Subpopulations in AMPV Vaccines Are Unlikely to Be the Only Cause of Reversion to Virulence. Vaccine.

[87] Beerens N., Heutink R., Pritz-Verschuren S., Germeraad E.A., Bergervoet S.A., Harders F., Bossers A., Koch G. (2019). Genetic Relationship between Poultry and Wild Bird Viruses during the Highly Pathogenic Avian Influenza H5N6 Epidemic in the Netherlands, 2017–2018. Transbound. Emerg. Dis..

[88] Pijnacker R., Dallman T.J., Tijsma A.S.L., Hawkins G., Larkin L., Kotila S.M., Amore G., Amato E., Suzuki P.M., Denayer S. (2019). An International Outbreak of Salmonella Enterica Serotype Enteritidis Linked to Eggs from Poland: A Microbiological and Epidemiological Study. Lancet Infect. Dis..

[89] Bali K., Kaszab E., Marton S., Hamdiou S.H., Bentaleb R.K., Kiss I., Palya V., Bányai K. (2022). Novel Lineage of Infectious Bronchitis Virus from Sub-Saharan Africa Identified by Random Amplification and Next-Generation Sequencing of Viral Genome. Life.

[90] Matos M., Bilic I., Viloux N., Palmieri N., Albaric O., Chatenet X., Tvarogová J., Dinhopl N., Heidl S., Liebhart D. (2022). A Novel Chaphamaparvovirus Is the Etiological Agent of Hepatitis Outbreaks in Pheasants (*Phasianus colchicus*) Characterized by High Mortality. Transbound. Emerg. Dis..

[91] Quince C., Walker A.W., Simpson J.T., Loman N.J., Segata N. (2017). Shotgun Metagenomics, from Sampling to Analysis. Nat. Biotechnol..

[92] Zhou Y., Liu M., Yang J. (2022). Recovering Metagenome-Assembled Genomes from Shotgun Metagenomic Sequencing Data: Methods, Applications, Challenges, and Opportunities. Microbiol. Res..

[93] Bogomolnaya L., Talamantes M., Rocha J., Nagarajan A., Zhu W., Spiga L., Winter M.G., Konganti K., Adams L.G., Winter S. (2023). Taxonomic and Metagenomic Analyses Define the Development of the Microbiota in the Chick. mBio.

[94] Wang Y., Lyu N., Liu F., Liu W.J., Bi Y., Zhang Z., Ma S., Cao J., Song X., Wang A. (2021). More Diversified Antibiotic Resistance Genes in Chickens and Workers of the Live Poultry Markets. Env. Int..

[95] Yan L., Chu T., Zhang Q., Blokker B., Lv Z., Geremia J., Bortoluzzi C. (2023). Microbiome Modulation by a Precision Biotic in Broilers Chickens: A Commercial Study Validation. Poult. Sci..

[96] Domingo E., Perales C. (2019). Viral Quasispecies. PLoS Genet..

[97] Correa-Fiz F., Franzo G., Llorens A., Huerta E., Sibila M., Kekarainen T., Segalés J. (2020). Porcine Circovirus 2 (PCV2) Population Study in Experimentally Infected Pigs Developing PCV2-Systemic Disease or a Subclinical Infection. Sci. Rep..

[98] Correa-Fiz F., Franzo G., Llorens A., Segalés J., Kekarainen T. (2018). Porcine Circovirus 2 (PCV-2) Genetic Variability under Natural Infection Scenario Reveals a Complex Network of Viral Quasispecies. Sci. Rep..

[99] Oade M.S., Keep S., Freimanis G.L., Orton R.J., Britton P., Hammond J.A., Bickerton E. (2019). Attenuation of Infectious Bronchitis Virus in Eggs Results in Different Patterns of Genomic Variation across Multiple Replicates. J. Virol..

[100] Legnardi M., Cecchinato M., Homonnay Z., Dauphin G., Koutoulis K.C., Tucciarone C.M., Franzo G. (2022). Viral Subpopulation Variability in Different Batches of Infectious Bronchitis Virus (IBV) Vaccines Based on GI-23 Lineage: Implications for the Field. Virus Res..

[101] Ndegwa E.N., Joiner K.S., Toro H., Van Ginkel F.W., Van Santen V.L. (2012). The Proportion of Specific Viral Subpopulations in Attenuated Arkansas Delmarva Poultry Industry Infectious Bronchitis Vaccines Influences Vaccination Outcome. Avian Dis..

[102] Zegpi R.A., Joiner K.S., Van Santen V.L., Toro H. (2019). Infectious Bronchitis Virus Population Structure Defines Immune Response and Protection. Avian Dis..

[103] Toro H., Pennington D., Gallardo R.A., van Santen V.L., van Ginkel F.W., Zhang J., Joiner K.S. (2012). Infectious Bronchitis Virus Subpopulations in Vaccinated Chickens After Challenge. Avian Dis..

[104] Faria N.R., Suchard M.A., Rambaut A., Lemey P. (2011). Toward a Quantitative Understanding of Viral Phylogeography. Curr. Opin. Virol..

[105] Baele G., Lemey P., Bedford T., Rambaut A., Suchard M.A., Alekseyenko A.V. (2012). Improving the Accuracy of Demographic and Molecular Clock Model Comparison While Accommodating Phylogenetic Uncertainty. Mol. Biol. Evol..

[106] Franzo G., Massi P., Tucciarone C.M., Barbieri I., Tosi G., Fiorentini L., Ciccozzi M., Lavazza A., Cecchinato M., Moreno A. (2017). Think Globally, Act Locally: Phylodynamic Reconstruction of Infectious Bronchitis Virus (IBV) QX Genotype (GI-19 Lineage) Reveals Different Population Dynamics and Spreading Patterns When Evaluated on Different Epidemiological Scales. PLoS ONE.

[107] Franzo G., Tucciarone C.M., Blanco A., Nofrarías M., Biarnés M., Cortey M., Majó N., Catelli E., Cecchinato M. (2016). Effect of Different Vaccination Strategies on IBV QX Population Dynamics and Clinical Outbreaks. Vaccine.

[108] Lemey P., Rambaut A., Drummond A.J., Suchard M.A. (2009). Bayesian Phylogeography Finds Its Roots. PLoS Comput. Biol..

[109] Franzo G., Tucciarone C.M., Moreno A., Legnardi M., Massi P., Tosi G., Trogu T., Ceruti R., Pesente P., Ortali G. (2020). Phylodynamic Analysis and Evaluation of the Balance between Anthropic and Environmental Factors Affecting IBV Spreading among Italian Poultry Farms. Sci. Rep..

[110] Holmes E.C., Grenfell B.T. (2009). Discovering the Phylodynamics of RNA Viruses. PLoS Comput. Biol..

[111] Rife B.D., Mavian C., Chen X., Ciccozzi M., Salemi M., Min J., Prosperi M.C. (2017). Phylodynamic Applications in 21st Century Global Infectious Disease Research. Glob. Health Res. Policy.

[112] Lemey P., Rambaut A., Bedford T., Faria N., Bielejec F., Baele G., Russell C.A., Smith D.J., Pybus O.G., Brockmann D. (2014). Unifying Viral Genetics and Human Transportation Data to Predict the Global Transmission Dynamics of Human Influenza H3N2. PLoS Pathog..

[113] Dellicour S., Rose R., Pybus O.G. (2016). Explaining the Geographic Spread of Emerging Epidemics: A Framework for Comparing Viral Phylogenies and Environmental Landscape Data. BMC Bioinform..

[114] Dellicour S., Lemey P., Suchard M.A., Gilbert M., Baele G. (2022). Accommodating Sampling Location Uncertainty in Continuous Phylogeography. Virus Evol..

[115] Dellicour S., Rose R., Faria N.R., Lemey P., Pybus O.G. (2016). SERAPHIM: Studying Environmental Rasters and Phylogenetically Informed Movements. Bioinformatics.

[116] Franzo G., Barbierato G., Pesente P., Legnardi M., Tucciarone C.M., Sandri G., Drigo M. (2021). Porcine Reproductive and Respiratory Syndrome (Prrs) Epidemiology in an Integrated Pig Company of Northern Italy: A Multilevel Threat Requiring Multilevel Interventions. Viruses.

[117] Franzo G., Tucciarone C.M., Cecchinato M., Drigo M. (2016). Porcine Circovirus Type 2 (PCV2) Evolution before and after the Vaccination Introduction: A Large Scale Epidemiological Study. Sci. Rep..

[118] Kosakovsky Pond S.L., Frost S.D.W. (2005). Not so Different after All: A Comparison of Methods for Detecting Amino Acid Sites under Selection. Mol. Biol. Evol..

[119] Pond S.L.K., Murrell B., Poon A.F.Y. (2012). Evolution of Viral Genomes: Interplay between Selection, Recombination, and Other Forces. Methods Mol. Biol..

[120] Murrell B., Scheffler K. (2012). Improved Models of Biological Sequence Evolution. Ph.D. Thesis.

[121] Franzo G., Legnardi M., Tucciarone C.M., Drigo M., Martini M., Cecchinato M. (2019). Evolution of Infectious Bronchitis Virus in the Field after Homologous Vaccination Introduction. Vet. Res..

[122] Posadas B.B., Gilbert J.E. (2020). Regulating Big Data in Agriculture. IEEE Technol. Soc. Mag..

[123] Carbonell I.M. (2016). The Ethics of Big Data in Big Agriculture. Internet Policy Rev..

[124] USAID U.S. (2022). Government Global Food Security Strategy 2022–2026.

[125] Krell N.T., Giroux S.A., Guido Z., Hannah C., Lopus S.E., Caylor K.K., Evans T.P. (2021). Smallholder Farmers’ Use of Mobile Phone Services in Central Kenya. Clim. Dev..

[126] Perez A.M., Linhares D.C.L., Arruda A.G., Van Der Waal K., Machado G., Vilalta C., Sanhueza J.M., Torrison J., Torremorell M., Corzo C.A. (2019). Individual or Common Good? Voluntary Data Sharing to Inform Disease Surveillance Systems in Food Animals. Front. Vet. Sci..

